# The Prevalence, Risk, and Management of Methicillin-Resistant *Staphylococcus aureus* Infection in Diverse Populations across Canada: A Systematic Review

**DOI:** 10.3390/pathogens10040393

**Published:** 2021-03-25

**Authors:** Elena Mitevska, Britney Wong, Bas G. J. Surewaard, Craig N. Jenne

**Affiliations:** 1 Immunology and Infectious Diseases, Department of Microbiology, University of Calgary, Calgary, AB T2N1N4, Canada; elena.mitevska@ucalgary.ca (E.M.); britney.wong@ucalgary.ca (B.W.); 2 Snyder Institute for Chronic Diseases, Department of Physiology and Pharmacology, University of Calgary, Calgary, AB T2N1N4, Canada; bgjsurew@ucalgary.ca

**Keywords:** methicillin-resistant *Staphylococcus aureus*, MRSA, prevalence

## Abstract

Methicillin-resistant *Staphylococcus aureus* (MRSA) first emerged after methicillin was introduced to combat penicillin resistance, and its prevalence in Canada has increased since the first MRSA outbreak in the early 1980s. We reviewed the existing literature on MRSA prevalence in Canada over time and in diverse populations across the country. MRSA prevalence increased steadily in the 1990s and 2000s and remains a public health concern in Canada, especially among vulnerable populations, such as rural, remote, and Indigenous communities. Antibiotic resistance patterns and risk factors for MRSA infection were also reported. All studies reported high susceptibility (>85%) to trimethoprim-sulfamethoxazole, with no significant resistance reported for vancomycin, linezolid, or rifampin. While MRSA continues to have susceptibility to several antibiotics, the high and sometimes variable resistance rates to other drugs underscores the importance of antimicrobial stewardship. Risk factors for high MRSA infection rates related to infection control measures, low socioeconomic status, and personal demographic characteristics were also reported. Additional surveillance, infection control measures, enhanced anti-microbial stewardship, and community education programs are necessary to decrease MRSA prevalence and minimize the public health risk posed by this pathogen.

## 1. Introduction

*Staphylococcus aureus* is part of the normal skin and nasal microbiota, and around 30% of the healthy adult population is colonized mainly in the nasopharyngeal cavity [1]. While colonization is usually asymptomatic, symptomatic infection can occur if there is a breach in the mucosal barrier or skin [1]. The severity of symptomatic infection ranges from superficial skin and soft tissue infections (SSTIs) to life threatening diseases, such as bacteremia or sepsis [1]. While many strains of *S. aureus* are easily treatable with a variety of antimicrobial compounds, methicillin-resistant *Staphylococcus aureus* (MRSA) presents a more challenging problem. MRSA first emerged in the United Kingdom after methicillin was introduced in hospitals to combat penicillin-resistant *S. aureus* in 1961, and MRSA incidence has increased since that time [2].

The first recorded outbreak of MRSA in Canada occurred in the early 1980s within a hospital setting [2]. In the early 2000s, infections arose in otherwise healthy community members and a distinction was made between hospital-acquired MRSA (HA-MRSA) and community-acquired MRSA (CA-MRSA) in Canada [3]. Not only do HA- and CA-MRSA differ with regards to the context in which these infections are acquired, these strains have also emerged with distinct genetic and phenotypic characteristics [1]. Notably, CA-MRSA strains can cause severe infections in otherwise healthy individuals. CA-MRSA strains display enhanced virulence, spreading more rapidly and causing more severe illness than HA-MRSA strains [4]. Enhanced virulence of CA-MRSA is thought to be associated with the production of several toxins, such as phenol-soluble modulins (PSMs) [4]. HA-MRSA strains contain the larger Staphylococcal cassette chromosome mec (SCCmec) types, I, II, or III, whereas CA-MRSA strains contain the smaller SCCmec types, IV or V [3]. The epidemiological criteria that was initially used to classify the strains has now become less useful as CA-MRSA has also moved into the hospital setting. Importantly, unlike in other parts of the world, the majority of MRSA infections in Canada remain attributable to healthcare exposure [3]. 

Given the changing dynamics of MRSA transmission, several surveillance programs have been implemented in Canada to track and monitor MRSA infections. The Canadian Nosocomial Infection Surveillance Program (CNISP) has monitored MRSA prevalence since 1995 by collecting data on all hospitalized patients with MRSA from 47 hospitals located in 9 provinces [3]. Additionally, the Canadian Ward Surveillance Study (CANWARD) tracks the antimicrobial susceptibility of clinical isolates from the Emergency Department (ED), Intensive Care Unit (ICU), out-patient clinics, and medical surgical wards in 15 hospitals located in 8 provinces [3]. From 2013 to 2017 alone, the CNISP identified and tracked over 10,000 MRSA infections in Canada [5]. 

With a limited number of effective antibiotics available, MRSA is a growing public health concern in Canada [6]. MRSA is a leading cause of nosocomial infections, particularly in critically ill patients. The SARS-CoV2 pandemic has overcrowded intensive care units, and patients on ventilators are at high risk for MRSA infections. Such infections increase the morbidity and mortality rates of patients and lead to higher care costs and longer hospital stays [7,8,9,10]. Vulnerable communities, such as Indigenous populations living on reserves, those living in isolated rural communities, and persons who inject drugs are at particular risk of MRSA infection due to limited or discriminatory access to healthcare, crowding, lack of sanitation equipment, and inadequate living conditions [11,12,13]. Accordingly, the purpose of this review is to describe the epidemiology of MRSA in Canada from 1991 to 2017, as well as the associated risk factors and intervention strategies.

## 2. Results

**Figure 1:**
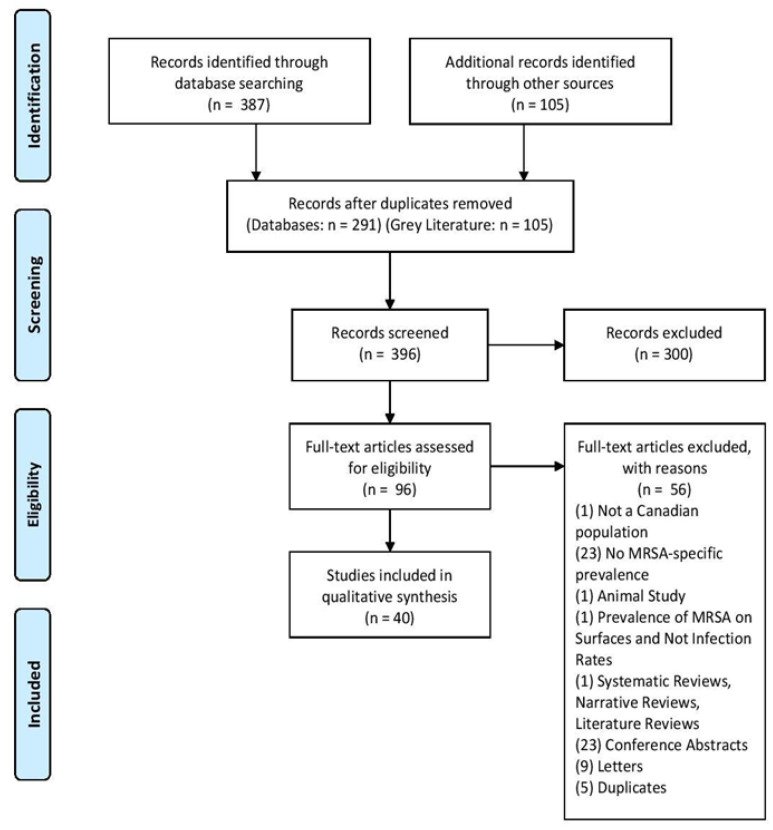
The Preferred Reporting Items for Systematic Reviews and Meta-Analysis (PRISMA) flow chart indicating the number of identified, screened, eligible, and included articles and the reasons for full-text exclusion.

A total of 387 citations were generated from the MEDLINE and EMbase searches. An additional 105 citations were obtained from the grey literature search. After removing duplicates, 396 articles remained for level-one screening. Level-one inclusion criteria consisted of a Canadian population and a mention of MRSA infection. These criteria resulted in the exclusion of 300 articles, while 96 citations proceeded to level two full-text screening. Subsequently, 56 full-text articles were excluded due to the reasons depicted in Figure 1. The principle reasons for exclusion were due to an absence of a full-text article (n = 23) or a lack of MRSA-specific prevalence rate (n = 23). In total, 40 articles were included for the qualitative analysis.

All data are summarized in Table A2, Table A3, Table A4 and Table A5. After the initial MRSA outbreak in Canada, MRSA prevalence has steadily increased from levels documented in the earliest reports. Allard et al. [14] reported no MRSA cases from 1991 to 1999 in a Quebec hospital, but prevalence rates increased to 2.6 per 100,000 patients from 2000 to 2002 and again to 7.4 per 100,000 patients from 2002 to 2005. Likewise, Simor et al. [8] found similarly increasing rates in hospitals across the country from 0.25 MRSA cases per 1000 admissions in 1995 to 1.11 cases per 1000 admissions in 1999, a more than 4-fold increase in less than half a decade. While still relatively low, Warshawsky et al. [15] reported a MRSA rate of 37 cases per 100,000 patients in hospitals and clinics in London, Ontario, in 1997, and Zoutman et al. [16] reported a MRSA prevalence of 2.0 per 1000 admission in acute care hospitals across the country in 1999. Additionally, Sligl et al. [9] reported a MRSA infection rate of 3.1 per 1000 ICU admissions from 1997 to 2005 in Edmonton, documenting an overall increasing trend within this hospital setting. 

Following the first incidence of CA-MRSA in Canada in the early 2000s, several studies have demonstrated a continued rise in MRSA prevalence and increased incidence rates in both clinical and non-clinical settings. Jones et. al [17] found a MRSA prevalence of 7.2% in 87 hospital sites across the country from 2000 to 2002. Golding et al. [13] found the prevalence of CA-MRSA to be 8.2 per 10,000 in Northern Saskatchewan communities in 2001, whereas Cowie et al. [18] reported a 5.39% MRSA prevalence in acute care institutions in Vancouver in 2002. Analyzing data from Calgary Laboratory Services, Gill et al. [19] found a rate of 22.2 per 100,000 people in 2004. Additionally, there was a prevalence of 3.7 cases per 10,000 patient days among patients in a Toronto hospital in 2003 [20]. 

Although the general trend found reports of increasing prevalence over time, a small number of studies reported lower MRSA prevalence rates. Li et al. [21] reported a MRSA prevalence of 0.32 per 100,000 Albertans in 2005, and Bracco et al. [22] reported a MRSA prevalence of only 1.1% among ICU patients in Montreal from 2002 to 2004 inclusive, suggesting that there may be some site-to-site variation in the detection or prevalence of MRSA.

At the beginning of the century, MRSA prevalence in vulnerable populations was similar to that observed in hospital patients, but this prevalence has undergone a marked increase in just a few years [23,24]. 

One study found a MRSA prevalence of 146–482 per 10,000 residents in three Northern communities from 2006 to 2008 [11]. Another study similarly reported a MRSA prevalence of 168.1 cases per 10,000 residents that was found in 2006 in rural and remote communities [12]. Gilbert et al. [24] reported an infection and colonization rate of 5.5% in 2005 among study participants from homeless shelters, needle exchange and detoxification programs, an inner-city medical clinic, and a corrections facility in Calgary. Similar observations were made by Al-Rawahi et al. [23], reporting a 7.4% MRSA prevalence among injection drug users in Vancouver in 2000 and a much higher MRSA prevalence of 18.6% in 2006. Expanding on these observations, one study found very high rates of MRSA SSTI infections in an inner-city emergency department in Vancouver: 54.8% of all SSTI patients were infected with MRSA in 2003 and 68% were infected in a follow-up study 21 months later [25]. Szakacs et al. [26] reported a MRSA prevalence of 4.5% among inner city shelter residents in 2006.

In addition to the vulnerable population, MRSA was also present in the general population, even in individuals who had not had any recent contact with the healthcare system. Hanselman et al. [27] found a 3.18% MRSA colonization rate among schoolteachers at a conference in Toronto in 2006. Surprisingly, in contrast, reported rates of MRSA among healthcare workers were low. Saito et al. [10] found that none of the employees at an emergency department in Toronto had MRSA in 2009, and Trépanier et al. [28] found only 1 medical resident colonized with MRSA in a Quebec City hospital. 

Critically, studies that examined MRSA prevalence over a longer period of time provide further evidence of an increasing trend in prevalence. Mitchell et al. [29] found the proportion of MRSA among all healthcare-associated infections across Canada to be 3.9% in 2002 and 8.5% in 2017. Similarly, there was an increasing trend in a Vancouver emergency department (ED) from 12.0 MRSA wound isolates per 10,000 ED visits in 2003 to 34.3 per 10,000 visits in 2011 [30]. This observed growth in MRSA prevalence appears to have stabilized in the early 2000s, but rates continued to remain high from 2006 to 2010 across the country [6,31,32,33,34,35,36,37,38,39]. 

Moving into the last decade, Ugarte Torres et al. [40] found a MRSA colonization rate of 1.4% in Calgary sexually transmitted infection (STI) and community clinics in 2014, and Gill et al. [19] reported a MRSA prevalence of 81 cases per 100,000 people using Calgary Laboratory Services data. Despite appearing to stabilize in the early 2000s, MRSA prevalence continues to be a public health concern, especially among vulnerable populations. El Eman et al. [41] found a MRSA prevalence of 3.0 per 100 long-term care home residents in Ontario in 2011. Remote and indigenous communities continue to be disproportionately impacted: Muileboom et al. [42] reported 2482 MRSA cases per 100,000 clinic, laboratory, and hospital patients in Northwestern Ontario in 2011, mainly from remote Indigenous communities. Similarly, Jeong et al. [13] found a 14.78% MRSA prevalence in skin and soft tissue infections from 2012 to 2013 among First Nations communities across 5 provinces, and although Li et al. [21] reported a generally lower MRSA prevalence in 2012 of 1.44 per 100,000 Albertans, they noted that the majority of cases were from Indigenous communities. 

**Figure 2:**
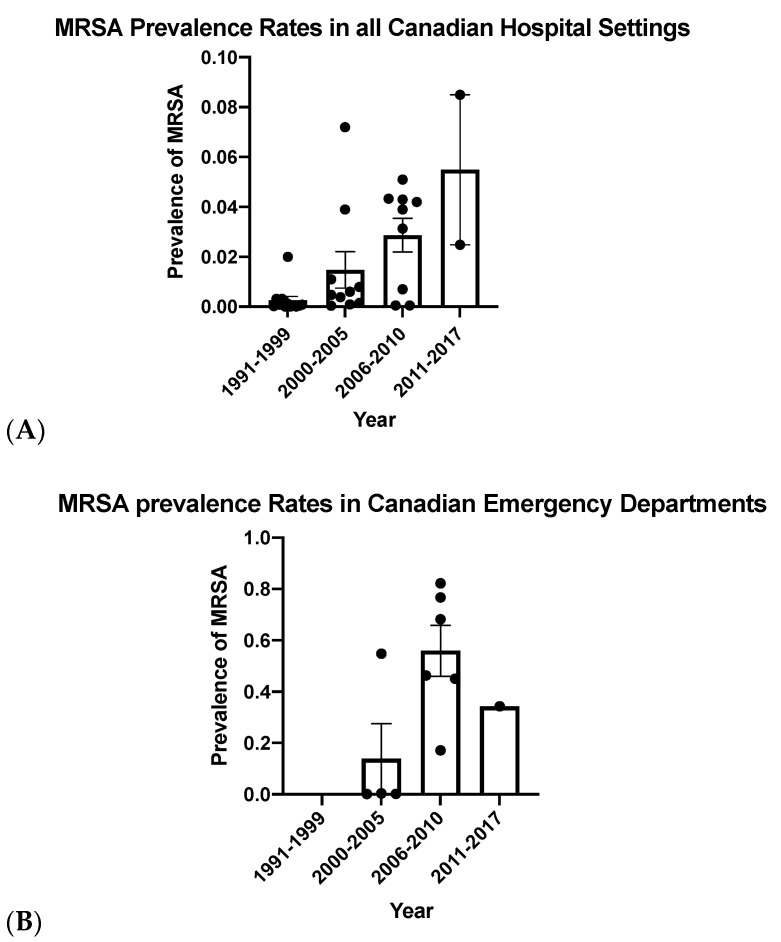
Methicillin-resistant *Staphylococcus aureus* (MRSA) prevalence rates compared in Canadian hospitals (**A**) and Canadian emergency departments (**B**) from 1991 until 2017. Bars represent mean prevalence rates and error bars represent the standard error of the mean.

Overall, increasing MRSA prevalence rates were observed in both hospitals and emergency departments (Figure 2), with higher prevalence seen in the emergency departments. Initially, epidemiological criteria were used to distinguish between CA-MRSA and HA-MRSA based on where the infection was acquired [3]. As previously discussed, multiple CA-MRSA strains have moved into the hospital setting, making this classification less useful [3]. Infections diagnosed in the emergency department can be used as a proxy for those acquired in the community, while infections diagnosed in the hospital are nosocomial if they have been diagnosed at least 48 h after admission. Given the higher prevalence of MRSA in the emergency department compared to hospital rates, it appears that MRSA infections occur more commonly in the community. 

## 3. Discussion

After the initial MRSA outbreak in the early 1980s in Canada, MRSA prevalence rose in the 1990s and early 2000s. MRSA rates appeared to have stabilized somewhat toward the end of the 2000s and into the 2010s, but MRSA remains a public health concern in Canada, especially among vulnerable populations, such as rural and remote and Indigenous communities. 

### 3.1. Antibiotic Resistance 

A subset of the studies cited also reported antibiotic resistance patterns in the identified MRSA isolates. Although all strains were resistant to beta-lactam antibiotics, such as penicillin, cloxacillin, and cefazolin [9,42], varying susceptibilities were reported for macrolide and lincosamide antibiotics that inhibit protein synthesis and for fluoroquinolones that disrupt DNA synthesis [9,31,39,42]. Sligl et al. [9] found that all strains were resistant to erythromycin (a macrolide) but 7% remained susceptible to clindamycin (a lincosamide) in a retrospective cohort analysis of MRSA infections in the intensive care unit at the University of Alberta hospital from 1997 to 2005. Kim et al. [43] similarly found complete resistance to erythromycin in MRSA infections in Alberta from 2005 to 2008. In contrast, Achiam et al [31] found that 7.4% of MRSA isolates from adult patients with SSTIs in an Ontario urban center were susceptible to erythromycin and 75% were susceptible to clindamycin, and Muileboom et al. [42] reported nearly 100% susceptibility to clindamycin and around 60% susceptibility to erythromycin in MRSA isolates from remote first nations communities from 2008 to 2012. Zhanel et al. [39] found resistance rates over 50% to fluoroquinolones and macrolides as part of the 2008 CANWARD study. Some of these differences in susceptibility rates might be due to the differing populations or locations of the studies, but they likely also indicate MRSA’s evolving and changing resistance patterns and the non-uniform distribution of resistance strains across the broad geography of Canada (urban vs. rural vs. Northern).

All studies reported high susceptibility (>85%) to the trimethoprim-sulfamethoxazole, indicating that this is a reasonable treatment for mild to moderate MRSA infections [9,39,42,43]. Additionally, none of the studies reported significant resistance to vancomycin, linezolid, or rifampin [9,31,39,43]. While MRSA continues to have susceptibility to several antibiotics, the high resistance and sometimes variable resistance rates to other drugs underscore the importance of antimicrobial stewardship. 

### 3.2. Risk Factors

Studies have reported various risk factors that result in higher MRSA infection rates. These risks are divided in several categories: infection control measures, factors affecting those with low socioeconomic status, and personal demographic characteristics. In the first category, multiple studies reported that large healthcare facilities have an increased risk of MRSA spread due to the high person-to-person contact. Within the ICU unit, Bracco et al. [22] suggest low compliance with hand hygiene as a risk for MRSA infection transmission. In addition, they warn that moving MRSA patients to different units before screening has been completed can cause cross-transmission in wards hyperendemic for MRSA. As a result, early identification and implementation of infection control measures must be strictly enforced within healthcare settings to prevent MRSA spread [15]. Within the community, MRSA risk factors included repeated injection drug use, having a previous MRSA infection, using antibiotics within the last 2 months and diabetes mellitus [25]. Furthermore, in two separate studies, Laupland et al. [44,45] identified that the male sex and older ages are factors at a higher risk for MRSA development.

The aforementioned MRSA prevalence rates indicated that vulnerable populations, such as remote Canadian Indigenous communities, are at a higher risk of developing MRSA infections. This is in part due to inadequate living conditions, such as crowded housing, poor access to running water, and a lack of sanitary supplies, which ultimately increase community transmission [13]. Specifically, Golding et al. [11] report that higher MRSA infection rates in remote Northern Saskatchewan communities are a result of overcrowding, poor housing conditions, inadequate hygiene, pre-existing skin conditions, and a high previous usage of antimicrobial drugs. Accordingly, Jeong et al. [13] suggests that developing a national-level surveillance system to monitor SSTI epidemiology and CA-MRSA antibiotic susceptibility test results would be essential for prevention, control, and management of disease spread, particularly in rural communities. Upstream factors of MRSA infection, including socio-environmental factors, such as poor housing and access to clean drinking water, must be addressed to prevent antibiotic resistance development and MRSA spread.

### 3.3. Future Directions

There are several interventions that can address MRSA spread and antibiotic resistance rates. First, while continuing current surveillance programs, more investment should be made in local, national, and international infection surveillance and control programs in both larger hospitals and remote communities. These efforts will help characterize the continuing evolution of MRSA colonization and infection patterns and their impact on patient outcomes [46]. Additional research is needed into MRSA transmission, risk factors, and infection control strategies, such as screening and decolonization. In particular, greater screening and contact precautions, such as improved hand hygiene compliance, should be adopted in hospitals to reduce nosocomial MRSA transmission [20]. Additionally, engineering controls should be explored. For example, decreasing patient contact by increasing the number of single-patient rooms has been shown to prevent MRSA transmission, encouraging us to rethink hospital and clinic design [22]. Moreover, further genetic characterization, virulence profiling, and detection of resistant determinants should be conducted to provide a better understanding of the biology, evolution, and adaptation of this clinically important pathogen.

Lastly, among vulnerable populations, educational tools have proven to be effective in combating MRSA infection rates. Golding et al. [12] introduced a “Germs Away” curriculum among Northern communities in Saskatchewan. This program, which featured proper handwashing techniques and cough etiquette, resulted in a decrease in MRSA rates in targeted communities over a 2-year period. This study demonstrates the promise of educational programs in schools and rural communities in mitigating CA-MRSA infection rates and the rates of other communicable diseases. 

## 4. Materials and Methods

A systematic review study protocol was created a priori and follows the Preferred Reporting Items for Systematic Reviews and Meta-Analysis (PRISMA) protocols, statements, and guidelines [47].

### 4.1. Search Strategy

MEDLINE and EMbase were searched from inception to 5 March 2020. Search clusters consisted of keywords and database-specific words. The keywords were used in both the MEDLINE and EMbase searches. The main clusters were “MRSA” terms, “Canada” terms, and “Prevalence” terms. The keywords and database-specific terms within each cluster were combined using “OR”, while each of the clusters were combined using “AND”. The search was conducted alongside an experienced librarian (Lee-Robertson, H., personal communication, 5 March 2020.) at the University of Calgary. A grey literature search was also conducted to augment the search on Google Scholar from inception to 30 March 2020, using only the keywords shown in Table A1. There were no language, age, or date restrictions for either the database searches or the grey literature search.

### 4.2. Eligibility Criteria and Selection of Articles

All articles were selected for eligibility and screened by two independent authors (B.W, E.M). To be included at the first stage screening process, each abstract needed to contain data on MRSA rates in a human population in Canada. All abstracts included at this first stage were eligible for screening at stage two. Screening by each author was performed separately and compared periodically to ensure that there was consistent agreeability between reviewers. Second stage screening consisted of the full-length text, and all included articles were assessed against the exclusion criteria outlined in Figure 1. Once again, each author independently screened the full texts at stage two, and each independent decision was compared. When the assessors disagreed on whether to include an abstract during the first or second stage of screening, a third-party adjudicator was brought in to help reach a decision of inclusion or exclusion. All articles that surpassed both stages were included at the data extraction stage.

### 4.3. Data Extraction Process and Synthesis of Evidence

Data was extracted in duplicate by two independent assessors. Data extraction criteria was formed together by both reviewers (B.W, E.M). The criteria were piloted using five randomly selected studies that were included after second-level screening. Both authors took part in extracting data from each included full-text, and the data was subsequently verified between authors.

## 5. Conclusions

MRSA infection remains a public health concern in Canada, especially among vulnerable populations. The risks associated with MRSA infections are multi-faceted and include personal demographic characteristics, infection control measures, and inadequate living conditions. Marginalized communities additionally face barriers to accessing timely and continuous medical care, highlighting inequities in our healthcare system. 

## Appendix A

**Table A1:** The systematic review search strategy within the MEDLINE and EMbase databases. MRSA terms, Canada terms, and prevalence terms were combined with “AND”, while keywords and database-specific terms within each search cluster were combined with “OR”.

Key Words		
MRSA Keywords	Canada Keywords	Prevalence Keywords
Methicillin-Resistant *Staphylococcus aureus*	Canada	Prevalence
MRSA	Canadian	Incidence
	Alberta	
	British Columbia	
	Manitoba	
	New Brunswick	
	Newfoundland	
	Labrador	
	Northwest Territories	
	Nova Scotia	
	Nunavut	
	Ontario	
	Prince Edward Island	
	Quebec	
	Saskatchewan	
	Yukon	
**Mesh Terms:**		
**MRSA Terms**	**Canada Terms**	**Prevalence Terms**
Methicillin-resistant *Staphylococcus Aureus*	Canada	Prevalence
	Alberta	Incidence
	British Columbia	
	Manitoba	
	New Brunswick	
	“Newfoundland and Labrador”	
	Northwest Territories	
	Nova Scotia	
	Nunavut	
	Ontario	
	Prince Edward Island	
	Quebec	
	Saskatchewan	
	Yukon Territory	
**EMtree Terms:**		
**MRSA Terms**	**Canada Terms**	**Prevalence Terms**
Methicillin-resistant *Staphylococcus aureus*	Canada	Epidemiological data
Meticillin resistant *Staphylococcus aureus*	Alberta	Prevalence
	British Columbia	Infection Rate
	Manitoba	Incidence
	New Brunswick	
	“Newfoundland and Labrador”	
	Northwest Territories	
	Nova Scotia	
	Nunavut	
	Ontario	
	Prince Edward Island	
	Quebec	
	Saskatchewan	
	Yukon	

**Table A2:** Prevalence of MRSA incidence, year(s) of data collection, region in Canada. and population from studies initiated between the years 1991 and 1999. All prevalence and incidence rates are infection rates unless otherwise specified.

First Author, Year	MRSA Prevalence	Year	Region	Population
Allard et al., 2008	(Bacteremia) Prevalence in 1991–1999: 0 per 100,000 2000–2002: 2.6 per 100,000 patients Prevalence in 2002–2005: 7.4/100,000	1991–2005	Quebec	Hospital Patients with at least 1 blood culture positive for *S. aureus*
Simor et al., 2001	Prevalence (per 1000 admissions): 1995: 0.25 1999:1.11	1995–1999	Across Canada	Hospital Patients
Simor et al., 2010	Prevalence (per 10,000 patient days): 1995: 0.36 2007: 3.43	1995–2007	Across Canada	Hospital Patients
Warshawsky et al., 2000	Prevalence: 37 per 100,000 patients	1997	London, ON	Hospital and Clinic Patients
Sligl et al., 2007	Infection Rate: 3.1 per 1000 ICU admissions (overall), Prevalence (per 1000 ICU admissions): 1997: 0.8 1998: 0.8 1999: 3.1 2000: 6.0 2001: 3.8 2002: 0.9 2003: 1.5 2004: 4.7 2005: 7.9	1997–2005	Edmonton, AB	ICU Patients
Zoutman et al., 2005	Prevalence: 2.0 per 1000 admissions	1999	Across Canada	Acute care hospital patients

**Table A3:** Prevalence of MRSA incidence, year(s) of data collection, region in Canada, and population from studies initiated between the years 2000 and 2005. All prevalence and incidence rates are infection rates unless otherwise specified.

First Author, Year	MRSA Prevalence	Year	Region	Population
Jones et. al, 2004	Prevalence: 7.20%	2000–2002	Across Canada	87 hospital sites
Al-Rawahi et al., 2008	Prevalence: 2000: 7.4% 2006: 18.6%	2000, 2006	Vancouver	Injection Drug Users
Golding et al., 2012	Prevalence: CA-MRSA 8.2/10,000	2001	Northern Saskatchewan	Northern Health Regions in Saskatchewan
Cowie et al., 2005	Prevalence: 22/408 participants = 5.39%	2002	Vancouver, BC	Acute Care Institutions
Bracco et al., 2007	Prevalence: 1.1%	2002–2004	Montreal	ICU patients
Mitchell et al., 2019	Prevalence (of all healthcare-associated infections): 2002: 3.9% 2017: 8.5%	2002, 2017	Across Canada	Hospital Inpatients
Poutanen et al., 2005	Prevalence: 3.7 per 10,000 patient days	2003	Toronto, ON	Hospital Patients
Stenstrom et al., 2009	Prevalence: 54.8% of SSTI patients, 68% after 21 months	2003–2004	Vancouver, BC	SSTI patients in ED
Wilmer et al., 2014	Prevalence (in wound isolates, per 10 000 ED visits): 2003: 12.0 2004: 23.0 2005: 40.1 2006: 76.7 2007: 82.2 2008: 68.2 2009: 45.0 2010: 46.3 2011: 34.3	2003–2011	Vancouver, BC	ED Patients
Gill et al., 2019	Prevalence: 22.2/100,000	2004	Calgary	Calgary Laboratory Services Data
Li et al., 2014	Prevalence: 0.32 per 100,000 people	2005	Alberta	Individuals living in Alberta
Gilbert et al., 2007	Prevalence (Infection and Colonization): 5.5% among 271 participants	2005	Calgary	From five study sites: an outreach needle-exchange van, homeless shelters, detoxification centres and residential substance treatment programs, and inner city medical clinic and new admissions to a local corrections facility

**Table A4:** Prevalence or incidence of MRSA, year(s) of data collection, region in Canada, and population from studies initiated between the years 2006 and 2010. All prevalence and incidence rates are infection rates unless otherwise specified.

First Author, Year	MRSA Prevalence or Incidence	Year	Region	Population
Hanselman et al., 2008	3.18% (7/220)	2006	Toronto	School Teachers in Toronto
Szakacs et al. 2007	Prevalence: 4.5%	2006	Ottawa, ON	Inner City Shelter Residents
Golding et al., 2011	Prevalence: 146–482/10,000	2006–2008	North Saskatchewan	3 Northern Saskatchewan communities
Roth et al., 2016	Prevalence: 46.79 per 100,000 patient days	2006–2009	Ottawa, ON	Hospital Patients
Li et al., 2017	Incidence Rate Ratio: 2006–2007: 1.022, April 2007–Jan 2009: 0.989, Jan 2010–Mar 2015: 0.987	2006–2015	Quebec	Teaching Facility Patients
Antoniou et al., 2009	Prevalence: Nasal or Rectal Carriage (CA-MRSA) = 1.6%	2007	Toronto	Mainly men who have sex with men
Adam et al., 2011	Prevalence: 356/8228	2007–2009	British Columbia, Alberta, Manitoba, Saskatchewan, Ontario, Quebec, New Brunswick, Nova Scotia	Canadian Hospitals
Golding et al., 2012	Prevalence (2006): 168.1 per 10,000 Prevalence (2008): 142.6 per 10,000	2006–2008	North Saskatchewan	Northern health regions in Saskatchewan
Achiam et al., 2011	Prevalence: 17.1% of all samples taken (13.2% from SSTI infection sites, 10.2% in nares cultures, 5.8% in throat cultures)	2008	London, Ontario	ED patients with SSTI infection
Zhanel et al., 2010	Prevalence: 5.1% of all bacterial isolates	2008	Across Canada	Hospital Patients
Wang et al., 2018	Prevalence of colonization: 3.13%	2008–2010	Ottawa, ON	Hospital Patients
Simor et al., 2016	Prevalence: 0.45 per 1000 admissions (MRSA BSI)	2008–2012	Across Canada	Hospital Patients
Saito et al., 2013	Prevalence: 0%	2009	Toronto, ON	ER Healthcare Workers
Trépanier et al., 2013	Prevalence (colonization): 0.4%	2010	Quebec City, QC	Medical Residents
Simor et al., 2013	Prevalence: 4.2% colonization, 0.3% infection rate	2010	All 10 Provinces	Hospital Inpatients
William et al., 2015	Prevalence: 2010: 4.3% 2012: 3.9%	2010, 2012	All 10 provinces	Hospital Patients
Martin et al., 2018	Prevalence: 0.7 per 100 inpatients	2010, 2012, 2016	Across Canada	Hospital Inpatients

**Table A5:** Prevalence of MRSA incidence, year(s) of data collection, region in Canada, and population from studies initiated after 2010. All prevalence and incidence rates are infection rates unless otherwise specified.

First Author, Year	MRSA Prevalence	Year	Region	Population
Eman et al., 2014	Prevalence: 3.0 cases per 100 residents	2011	Ontario	Long-term Care Homes in Ontario
Muileboom et al., 2013	Prevalence: 2482 per 100,000 patients	2011	NW Ontario	Clinic, Laboratory and Hospital Patients (mainly remote Indigenous communities)
Li et al., 2014	Prevalence: 1.44 per 100,000 people	2012	Alberta	Alberta Residents, most infected were Indigenous
Jeong et al., 2020	Prevalence: 14.78% (55/372)	2012–2013	AB, SK, MB, ON, QC	Individuals in First Nations Communities Across 5 Provinces with SSTIs
Ugarte Torres et al., 2017	Prevalence (colonization): 1.4%	2014	Calgary, AB	STI and Community Clinic Patients
Gill et al., 2019	Prevalence: 81/100,000	2014	Calgary	Calgary Laboratory Services Patients
